# *NBS1* I171V variant underlies individual differences in chromosomal radiosensitivity within human populations

**DOI:** 10.1038/s41598-021-98673-7

**Published:** 2021-10-04

**Authors:** Keita Tomioka, Tatsuo Miyamoto, Silvia Natsuko Akutsu, Hiromi Yanagihara, Kazumasa Fujita, Ekaterina Royba, Hiroshi Tauchi, Takashi Yamamoto, Iemasa Koh, Eiji Hirata, Yoshiki Kudo, Masao Kobayashi, Satoshi Okada, Shinya Matsuura

**Affiliations:** 1 Department of Genetics and Cell Biology, Research Institute for Radiation Biology and Medicine, Hiroshima University, Hiroshima, 734-8553 Japan; 2 Department of Pediatrics, Graduate School of Biomedical and Health Sciences, Hiroshima University, Hiroshima, 734-8551 Japan; 3 Center for Radiological Research, Columbia University Irving Medical Center, New York, 10032 USA; 4 Department of Biological Sciences, Faculty of Science, Ibaraki University, Mito, 310-8512 Japan; 5 Program of Mathematical and Life Sciences, Graduate School of Integrated Sciences for Life, Hiroshima University, Higashi-Hiroshima, 739-8526 Japan; 6 Department of Obstetrics and Gynecology, Graduate School of Biomedical and Health Sciences, Hiroshima University, Hiroshima, 734-8551 Japan

**Keywords:** Biotechnology, Cancer, Cell biology, Genetics, Risk factors

## Abstract

Genetic information is protected against a variety of genotoxins including ionizing radiation (IR) through the DNA double-strand break (DSB) repair machinery. Genome-wide association studies and clinical sequencing of cancer patients have suggested that a number of variants in the DNA DSB repair genes might underlie individual differences in chromosomal radiosensitivity within human populations. However, the number of established variants that directly affect radiosensitivity is still limited. In this study, we performed whole-exome sequencing of 29 Japanese ovarian cancer patients and detected the *NBS1* I171V variant, which is estimated to exist at a rate of approximately 0.15% in healthy human populations, in one patient. To clarify whether this variant indeed contributes to chromosomal radiosensitivity, we generated *NBS1* I171V variant homozygous knock-in HCT116 cells and mice using the CRISPR/Cas9 system. Radiation-induced micronucleus formation and chromosomal aberration frequency were significantly increased in both HCT116 cells and mouse embryonic fibroblasts (MEFs) with knock-in of the *NBS1* I171V variant compared with the levels in wild-type cells. These results suggested that the *NBS1* I171V variant might be a genetic factor underlying individual differences in chromosomal radiosensitivity.

## Introduction

Ionizing radiation (IR) induces DNA double-strand breaks (DSBs). When DNA DSBs are left unrepaired, such damage results in the loss or rearrangement of genomic information, leading to cell death or carcinogenesis. To maintain genomic integrity, mammalian cells respond to DNA DSBs through a variety of pathways, including DNA repair, cell cycle checkpoint, and apoptosis^1,2^. It has been shown that, within human populations, there are individual differences in the capacity of cells to repair DNA DSBs, which we define as chromosomal radiosensitivity in this paper. The term “radiosensitivity” is used to describe different events in irradiated cells. In general, “cellular radiosensitivity” is defined as the cellular lethality post-irradiation underlying the occurrence of acute radiation-induced tissue damage, whereas “chromosomal radiosensitivity,” which can be evaluated by several cytogenetic assays, is thought to be associated with the susceptibility to developing radiation-induced cancer^3,4^.

The cytokinesis-blocked micronucleus (CBMN) assay^5^, which is an elaborate procedure to evaluate chromosomal radiosensitivity by counting micronuclei formed by unrepaired DSB-derived chromosomal fragments, demonstrated the existence of mildly radiosensitive cases within a small population of healthy individuals^6^. It has been shown that DNA DSB repair gene variants might be the cause of individual differences in chromosomal radiosensitivity. To determine whether variants of DNA repair genes are involved in individual differences in radiosensitivity, it is informative to measure the chromosomal radiosensitivity of primary cells carrying the genetic variants of interest. However, chromosomal radiosensitivity might be influenced by many confounding factors, such as age, smoking, environment, and other genetic background^7^. It is therefore necessary to evaluate the genetic variants underlying individual differences in chromosomal radiosensitivity in a cell line with a uniform genetic background.

We previously reported genome editing technology-mediated candidate variant knock-in in human cultured cells with a uniform genetic background to confirm that heterozygous *ATM* mutations, which cause a rare autosomal-recessive disease, ataxia-telangiectasia [A-T; Online Mendelian Inheritance in Man (OMIM): 067585], indeed underlie chromosomal radiosensitivity^8^. Since heterozygous carriers of most hyper-radiosensitive recessive disorders such as A-T exist at a rate of ~ 1% in human populations^9^, it was suggested that the heterozygous mutations might also determine individual differences in chromosomal radiosensitivity within human populations.

The association between increased chromosomal radiosensitivity and sporadic breast cancer risk has been reported^6^, and a number of DNA DSB repair genes including *BRCA1*, *BRCA2*, *ATM*, *CHEK2*, *PALB2*, *TP53*, *Rad51C*, *Rad51D*, and *NBS1* have been identified as being causative of hereditary breast and ovarian cancer (HBOC)^10^. On the basis of this background, multi-gene panel testing of the DNA repair genes is clinically used as a tool for screening breast or ovarian cancer patients. In this study, we searched for variants in DNA DSB repair genes using whole-exome sequencing of 29 Japanese ovarian cancer patients. The heterozygous *NBS1* c.511A>G, p.Ile171Val variant was identified as a unique candidate underlying chromosomal radiosensitivity. It was reported that a Japanese girl with a homozygous variant of *NBS1* I171V developed idiopathic aplastic anemia, but did not show the typical features of hyper-radiosensitive and cancer-prone Nijmegen breakage syndrome [OMIM: 602667.0007]^11^. Meta-analysis based on 60 publications with ~ 40,000 cancer cases and ~ 65,000 controls demonstrated that the *NBS1* I171V variant is associated with a significant increase in overall cancer risk^12^, while several epidemiological studies did not show any contribution of this variant to carcinogenesis^13–16^. Given these conflicting interpretations of pathogenicity, we decided to analyze the exact functional impact of this variant on chromosomal radiosensitivity in a uniform genetic background. Here, we generated *NBS1* variant knock-in human cultured cells and MEFs using genome editing technology. Semiautomated CBMN and chromosome aberration analyses in both genome-edited human and mouse cells could quantify the effect of this variant on chromosomal radiosensitivity. These findings could provide a unique insight into the genetic basis underlying the heterogeneity of chromosomal radiosensitivity within human populations.

## Results

### Identification of *NBS1* I171V variant in a Japanese ovarian cancer patient and generation of knock-in HCT116 cell clones using genome editing technology

**Figure 1:**
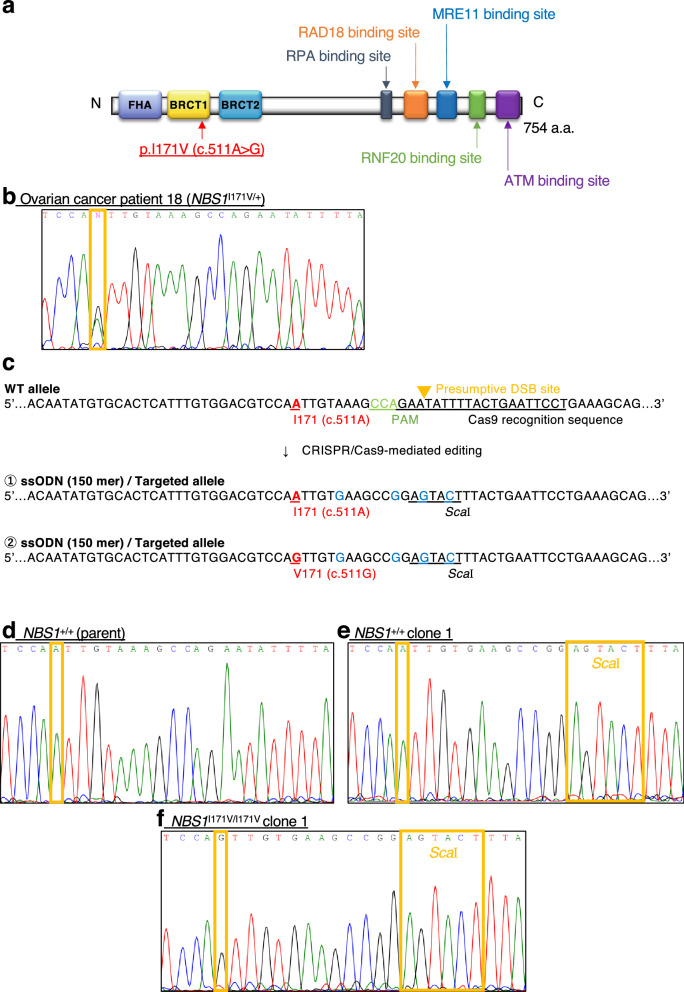
Generation of *NBS1* I171V knock-in HCT-116 cells. (**a**) Structure of human NBS1 protein. NBS1 contains FHA and BRCT1/2 domains at the N terminus and several DNA repair protein-binding regions at the C terminus. FHA and BRCT1/2 domains are involved in the IR-induced nuclear foci with phosphoproteins such as γ-H2AX. NBS1 I171V is located in the BRCT1 domain. (**b**) Sanger sequencing confirmed *NBS1* I171V heterozygosity in ovarian cancer patient 18. (**c**) Targeting strategy for *NBS1* I171V knock-in using the CRISPR/Cas9 system. Blue bases indicate silent mutations. (**d**–**f**) Sanger sequencing of *NBS1*^+/+^ parental HCT116 cell (**d**), *NBS1*^+/+^-edited HCT116 cell clone 1 (**e**), *NBS1*^I171V/I171V^-edited HCT116 cell clone 1 (**f**). A single base substitution of the *NBS1* I171V variant and a silent Sca I site are indicated by yellow boxes.

To screen genetic variants underlying chromosomal radiosensitivity, we performed whole-exome sequencing of genomic DNA from the peripheral blood cells of 29 Japanese ovarian cancer patients. Average coverage for the exons was more than 100 ×. Since most of the mutations detected in HBOC patients are located in the DNA repair genes*,* we first extracted genetic variants in the top 10 HBOC genes, namely, *BRCA1*, *BRCA2*, *ATM*, *CDH1*, *CHEK2*, *PALB2*, *TP53*, *Rad51C*, *Rad51D*, and *NBS1*^10^. We also narrowed down the candidate variants on the basis of filtering criteria consisting of the ClinVar database evaluation, genomic position, function, and zygosity. As expected, of these patients, five cases harbored heterozygous mutations of either *BRCA1* or *BRCA2* (Table 1). In addition, heterozygous *ATM* missense variants (rs551411717 and rs587782298) in two ovarian cancer patients and a heterozygous TP53 missense variant (rs201382018) in two patients, which might be causative mutations of ataxia-telangiectasia (A-T, OMIM: 607585) and Li-Fraumeni syndrome (OMIM: 191170), respectively, were detected (Table 2). We also identified heterozygous variant c.511A>G, p.Ile171Val, in the *NBS1* gene (NM: 002485.5, OMIM: 602667.0007), which encodes a component of the MRE11–RAD50–NBS1 (MRN) complex sensing DNA DSB sites for appropriate repair^17^, in one patient (Table 2, Fig. 1a,b). The *NBS1* c.511A>G, p.Ile171Val variant was predicted to be “disease-causing” and “probably damaging” by MutationTaster and Polyphen2, respectively. In addition, it was previously reported that a Japanese homozygote of this variant showed aplastic anemia rather than the typical features of Nijmegen breakage syndrome, such as severe microcephaly, immunodeficiency, and cancer predisposition^11^. These findings suggested that the *NBS1* c.511A>G, p.Ile171Val variant might be involved in individual differences in chromosomal radiosensitivity within human populations.

**Table 1:** Nonsense or frameshift mutations in the HBOC core gene identified by whole-exome sequencing in 29 ovarian cancer patients.

Gene	Variant ID	Nucleic acid change	Amino acid change	Allele frequency (gnomAD)	ClinVar	Mutation-taster	Ovarian cancer patient
*BRCA1*	rs80357692	c.3329_3330insA	p.Lys1110Gln1111fs	0.00001199	n.d.	Disease causing	3
	rs80357526	c.1953_1956delGAAA	p.Lys651fs	0.00000657	Pathogenic	Disease causing	6
	rs80357661	c.2767_2770delGTTA	p.Val923fs	0.000006572	Pathogenic	Disease causing	28
*BRCA2*	n.d.	c.1314_1315delTT	p.Asp438fs	n.d.	n.d.	Disease causing	8
	rs80358920	c.6952C>T	p.Arg2318*	n.d.	Pathogenic	Disease causing	10

**Table 2:** Missense variants in the HBOC core gene identified by whole-exome sequencing in 29 ovarian cancer patients.

Gene	Variant ID	Nucleic acid change	Amino acid change	Allele frequency (gnomAD)	ClinVar	Mutation-taster	Polyphen-2 (score)	Ovarian cancer patient
*BRCA1*	rs1597830733	c.4900A>G	p.Arg1634Gly	n.d.	Uncertain significance	Polymorphism	BENIGN (0.081)	1, 2*, 3, 4*, 5, 6, 7, 8*, 9*, 11, 12*, 13, 15, 16*, 17, 18, 19*, 20, 25*, 27, 28
	rs16942	c.3548A>G	p.Lys1183Arg	0.3486	Uncertain significance	Polymorphism	BENIGN (0)	1, 2*, 3, 4*, 5, 6, 7, 8*, 9*, 11, 12*, 13, 15, 16*, 17, 18, 19*, 20, 22, 23*, 25*, 27, 28
	rs80357244	c.811G>A	p.Val271Met	0.00009232	Conflicting interpretations of pathogenicity	Polymorphism	POSSIBLY DAMAGING (0.879)	4
	n.d.	c.1231G>C	p.Asp411His	n.d.	Uncertain significance	Polymorphism	BENIGN (0.043)	20
*ATM*	rs551411717	c.8288G>A	p.Arg2763Gln	0.00001972	Uncertain significance	Disease causing	PROBABLY DAMAGING (0.994)	13
	rs55870064	c.4949A>G	p.Asn1650Ser	0.0005629	Benign/Likely benign	Polymorphism	BENIGN (0)	21
	rs587782298	c.2771G>A	p.Arg924Gln	0.000007556	Uncertain significance	Disease causing	POSSIBLY DAMAGING (0.522)	25
*PALB2*	rs152451	c.1676A>G	p.Gln559Arg	0.1072	Benign/Likely benign	Polymorphism	BENIGN (0)	2, 3, 4, 7, 13, 14, 15, 17*, 18*, 19, 26, 29*
	rs756778249	c.1540G>A	p.Gly514Arg	0.00001315	Conflicting interpretations of pathogenicity	Polymorphism	BENIGN (0.145)	10
	rs141749524	c.2228A>G	p.Tyr743Cys	0.00007777	Conflicting interpretations of pathogenicity	Polymorphism	BENIGN (0)	26
*TP53*	rs201382018	c.31G>C	p.Glu11Gln	0.00000709	Conflicting interpretations of pathogenicity	Polymorphism	PROBABLY DAMAGING (0.996)	1, 26
*RAD51D*	rs56026142	c.196G>A	p.Val66Met	0.0003113	Conflicting interpretations of pathogenicity	Polymorphism	POSSIBLY DAMAGING (0.476)	14
*NBS1*	rs192236678	c.1809C>A	p.Phe603Leu	0.0001175	Conflicting interpretations of pathogenicity	Polymorphism	BENIGN (0)	7
	rs61754966	c.511A>G	p.Ile171Val	0.0015	Conflicting interpretations of pathogenicity	Disease causing	PROBABLY DAMAGING (1)	18

Asterisks indicate homozygosity of the variants. Variants with a “benign” status as evaluated in the ClinVar database are not included in this table.

**Figure 2:**
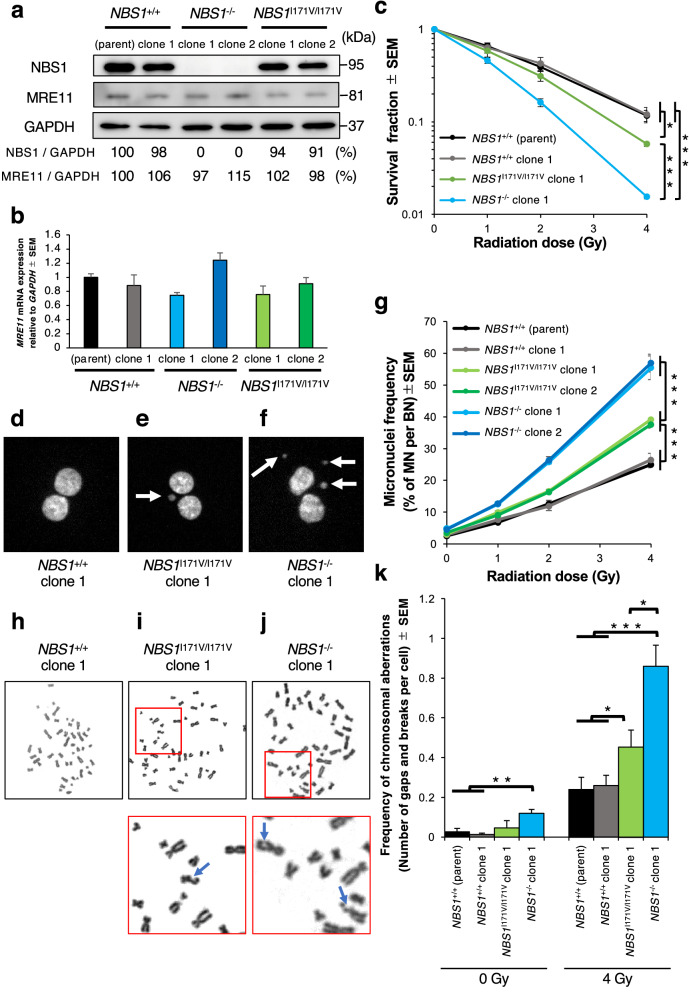
Chromosomal radiosensitivity of *NBS1* I171V edited HCT116 cell clones. (**a**) Western blotting analysis data showing expression levels of NBS1 and MRE11 protein in *NBS1* I171V edited HCT116 cell clones. Full-length gel is presented in Supplementary Fig. 5. GAPDH antibody was used as a loading control. The intensity of *NBS1* and *MRE11* bands was normalized to that of GAPDH and shown as a percentage, regarding the score of *NBS1*^+/+^ parent cell clones as 100%. (**b**) RT-PCR data showing mRNA expression levels of *MRE11* in *NBS1* I171V edited HCT116 cell clones. *GAPDH* was used as a control. (mean ± SEM; no significant change in each *t*-test parameter; n = 4) (**c**) Survival fractions for *NBS1* I171V edited HCT116 cell clones for 11 days after irradiation (mean ± SD based on averages from triplicate samples; *t*-test; n = 3). (**d**–**f**) Metafer MN Search images showing the cytokinesis-blocked *NBS1* I171V edited HCT116 cells stained with DAPI. Arrowheads indicate MN. BN cell without MN of *NBS1*^+/+^ clone 1 (**d**); BN cell with one MN of *NBS1*^I171V/I171V^ clone 1 (**e**); BN cell with three MN of *NBS1*^−/−^ clone 1 (**f**). (**g**) Percentage of IR-induced MN formation in *NBS1* I171V edited HCT116 cell clones (mean ± SEM; *t*-test; n = 3; > 1000 BN cells). (**h**–**j**) Representative metaphase after 4 Gy irradiation of *NBS1* I171V edited HCT116 cells. Remarkable aberrations are enlarged. Arrows indicate chromosomal breakages. Metaphase without chromosomal breakages of *NBS1*^+/+^ clone 1 (**h**), metaphase with one chromosomal breakage of *NBS1*^I171V/I171V^ clone 1 (**i**), and metaphase with two chromosomal breakages of *NBS1*^−/−^ clone 1 (**j**). (**k**) Frequency of IR-induced chromosomal aberration in *NBS1* I171V edited HCT116 cell clones (mean ± SEM; *t*-test; n = 3; > 50 cells).

To demonstrate that the *NBS1* c.511A>G, p.Ile171Val variant indeed underlies chromosomal radiosensitivity, we attempted to generate cultured human cells with knock-in of this variant along with a uniform genetic background using the CRISPR/Cas9 system. We constructed a plasmid vector expressing both Cas9 protein and single guide RNA (sgRNA) for cutting *NBS1* exon 5 including c.511A>G, p.Ile171Val (Fig. 1c). The plasmid vector (px459 provided by Addgene) contained the *Cas9* gene and a puromycin resistance gene separated by a 2A peptide sequence, expressing the discrete proteins from a single open reading frame. As a targeting donor, we chemically synthesized single-strand oligonucleotides (ssODNs) harboring the *NBS1* c.511A>G, p.Ile171Val variant and putative silent CRISPR/Cas9 blocking mutations in the PAM and sgRNA sequences (Fig. 1c). These silent mutations also functioned as an *Sca*I site for checking ssODN knock-in easily (Fig. 1c). We also designed ssODNs carrying the silent mutations alone in order to evaluate their effect on chromosomal radiosensitivity (Fig. 1c). Since the human colon cancer cell line HCT116 has two copies of the *NBS1* allele and relatively high efficacy of ssODN knock-in^18,19^, we transfected both the Cas9-2A-puromycin resistance gene plasmid vector and the ssODN targeting donors into HCT116 cells. After transient puromycin selection for 48 h post-transfection and subsequent culture for 2 weeks, the drug-resistant colonies were isolated, and their genotypes were analyzed by *Sca*I digestion and direct sequencing of the PCR amplicon of the target locus. Finally, two clones of the biallelic *NBS1* c.511A>G, p.Ile171Val knock-in cells (*NBS1*^I171V/I171V^ cells) and one clone of the biallelic wild-type *NBS1* with silent mutation knock-in cells (*NBS1*^+/+^ cells) were generated (Fig. 1d–f). We also established two clones of the *NBS1* null HCT116 cells (*NBS1*^−/−^ cells) with c.514_514insG, p.Val172fs and c.660_6678del, p.Gln220fs mutations. Western blotting analysis revealed that the *NBS1*^−/−^ clones had no signal of NBS1 protein, while the *NBS1*^I171V/I171V^ clones showed almost the same amounts of NBS1 protein as the *NBS1*^+/+^ parental HCT116 cells and *NBS1*^+/+^ cell clone, suggesting that the *NBS1* c.511A>G, p.Ile171Val variant is not involved in the stability of NBS1 protein (Fig. 2a). It was reported that over extended culturing of HCT116 cells caused mutations in the *MRE11* promoter thereby causing loss of expression of *MRE11*^20^. These generated clones showed almost the same amounts of MRE11 protein and mRNA level (Fig. 2a,b). Thus, CRISPR/Cas9 system-mediated knock-in technology in the HCT116 cell line enabled the generation of an experimental system for comparing the biological effects among the *NBS1* variants with a uniform genetic background.

### Chromosomal radiosensitivity is more enhanced in *NBS1*^I171V/I171V^-edited HCT116 cell clones than in *NBS1*^+*/*+^ HCT116 cells

To investigate whether the *NBS1* c.511A>G, p.Ile171Val variant affects the cellular lethality after γ-ray irradiation, we performed the colony survival assay of the set of *NBS1* genome-edited cell clones. Consistent with previous reports^21,22^, the *NBS1*^−/−^ clone showed much higher lethal radiosensitivity than the others. The *NBS1*^I171V/I171V^ cell clone did not show significant cellular lethality after less than 2 Gy of γ-ray irradiation in comparison with the *NBS1*^+/+^ cell line (Fig. 2c, S3a–c, Table S1), while their lethal radiosensitivity was clearly segregated from those of the *NBS1*^+/+^ cell line in the higher dose exposure. These results implied that the *NBS1* c.511A>G, p.Ile171Val variant might contribute to cellular radiosensitivity.

Next, to quantify effect of the *NBS1* c.511A>G, p.Ile171Val variant on radiosensitivity at the chromosomal level, radiation-induced micronuclei (MN) in the binucleated (BN) cells were measured (Fig. 2d–f). We evaluated the average ratio of MN to BN cells scored on more than 1000 BN images acquired from Metafer system as a semiautomatic approach^8^, in three independent conditions along with the standard error for each point and create representative dose–response calibration curves. The *NBS1*^−/−^ cell clones showed extreme IR-induced micronucleus formation, while the *NBS1*^I171V/I171V^ cell clones demonstrated a highly radiosensitive phenotype in comparison with the *NBS1*^+/+^ parental HCT116 cell line and *NBS1*^+/+^ cell clone with silent mutations (Fig. 2g, S3d–f, Table S2). To compare the chromosomal radiosensitivity among these cell clones in a more quantitative manner, we used a linear-quadratic model (MN frequency = c + βD + αD^2^, D: exposure dose) to analyze the dose–response curves of the ratio of MN/BN cells among the *NBS1*-edited HCT116 cell clones. α, β, and c coefficients were scored by the chromosomal aberration calculation (Cabas) software version 2.0 (http://www.pu.kielce.pl/ibiol/cabas)^23^. Since a linear-quadratic model is converted to (MN frequency-c)/D = αD + β, the sum of α and β coefficients at D = 1 Gy accurately represents the radiosensitivity of cells to γ-ray irradiation^24^. The mean data scores of the sum of α and β (D = 1 Gy) from the *NBS1*-edited HCT116 cell clones were obtained (Table 3), indicating that biallelic *NBS1* null mutations and the c.511A>G, p.Ile171Val variant contributed to approximately 2.0- and 1.3-fold increases of chromosomal radiosensitivity, respectively, in the HCT116 cell genetic background.

**Table 3:** Radiosensitivity coefficients (α, β, c, and α + β) in *NBS1* I171V variant knock-in HCT116 cells.

Cell line ID/genotype	β ± SE × 10^–3^	α ± SE × 10^–3^	c ± SE × 10^–3^	Radiosensitivity score [α + β]
	(Gy^-2^)	(Gy^-1^)		
*NBS1*^+/+^ (parent)	3.736 ± 1.10	41.51 ± 4.87	24.99 ± 2.12	45.25 ± 4.03
*NBS1*^+/+^ clone 1	5.407 ± 1.59	36.77 ± 5.80	29.02 ± 2.56	42.17 ± 4.74
*NBS1*^I171V/I171V^ clone 1	9.027 ± 0.85	53.23 ± 4.32	31.38 ± 3.78	62.25 ± 3.59
*NBS1*^I171V/I171V^ clone 2	10.34 ± 1.35	43.57 ± 3.34	35.81 ± 3.24	53.91 ± 2.49
*NBS1*^−/−^ clone 1	13.77 ± 2.92	72.83 ± 2.87	46.67 ± 0.83	86.60 ± 0.76
*NBS1*^−/−^ clone 2	14.00 ± 2.72	76.05 ± 2.77	44.74 ± 3.81	90.05 ± 0.11

α, β, and c coefficients were extracted from dose–response calibration curves in the CBMN assay using Cabas software. Relative capacity to repair DNA after acute γ-irradiation was assessed at a dose of 1 Gy.

To confirm the radiosensitivity of the *NBS1* c.511A>G, p.Ile171Val variant at the chromosomal level more directly, we stained the chromosomes with Giemsa dye in the γ-ray-irradiated *NBS1* edited cell lines, and then measured the chromosomal aberrations including chromatid and chromosome gaps and breaks^25^. Consistent with the results of the semiautomatic CBMN assay, the *NBS1*^−/−^ cells showed the highest ratio of radiation-induced chromosomal aberrations, while the *NBS1*^I171V/I171V^ cells demonstrated an incidence of them intermediate between those of the *NBS1*^−/−^ cells and the *NBS1*^+/+^ cell clones (Fig. 2h–k, S3g–i, Table S3). These findings revealed that the *NBS1* c.511A>G, p.Ile171Val variant has potency for enhancing chromosomal radiosensitivity.

### Chromosomal radiosensitivity increases in a manner dependent on the copy number of *Nbs1* I171V variant

We attempted to generate heterozygous *NBS1*^I171V/+^ HCT116 cell clones using the genome editing method mentioned above. However, the candidate cell clones obtained were all genetically mosaics consisting of *NBS1*^+/+^ and *NBS1*^I171V/ I171V^ cells, and no heterozygous *NBS1*^I171V/+^ single-cell clones were isolated.

Using genome editing technology in mouse fertilized embryos, it is possible to establish heterozygous cell clones for the ssODN knock-in allele. We thus attempted to establish *Nbs1* I171V knock-in mice by co-electroporation of CRISPR/Cas9 RNP, which constitutes recombinant Cas9 protein, chemically synthesized crRNA and tracRNA, and ssODN containing the variant, into mouse fertilized eggs (Fig S1a). Two mouse lines were generated from distinct electroporated embryos, in which the *Nbs1* p.Ile171Val variant was introduced into the genome. Sanger sequencing confirmed correct knock-in of the variant in both lines. Since the two mouse lines did not exhibit different phenotypes, we mainly addressed data obtained from one line.

No obvious phenotypes were observed in heterozygous mutant mice up to 1 year of age. When they were inbred, wild-type (*Nbs1*^+/+^), heterozygous (*Nbs1*^I171V/+^), and homozygous (*Nbs1*^I171V/I171V^) mice were born at the expected Mendelian ratio. *Nbs1*^I171V/I171V^ mice developed and grew normally in the laboratory environment and showed normal reproductive ability, and did not show any hematological profile such as aplastic anemia, which was previously reported in a human homozygote of the *NBS1* c.511A>G, p.Ile171Val variant^11^ (Fig. S2).

**Figure 3:**
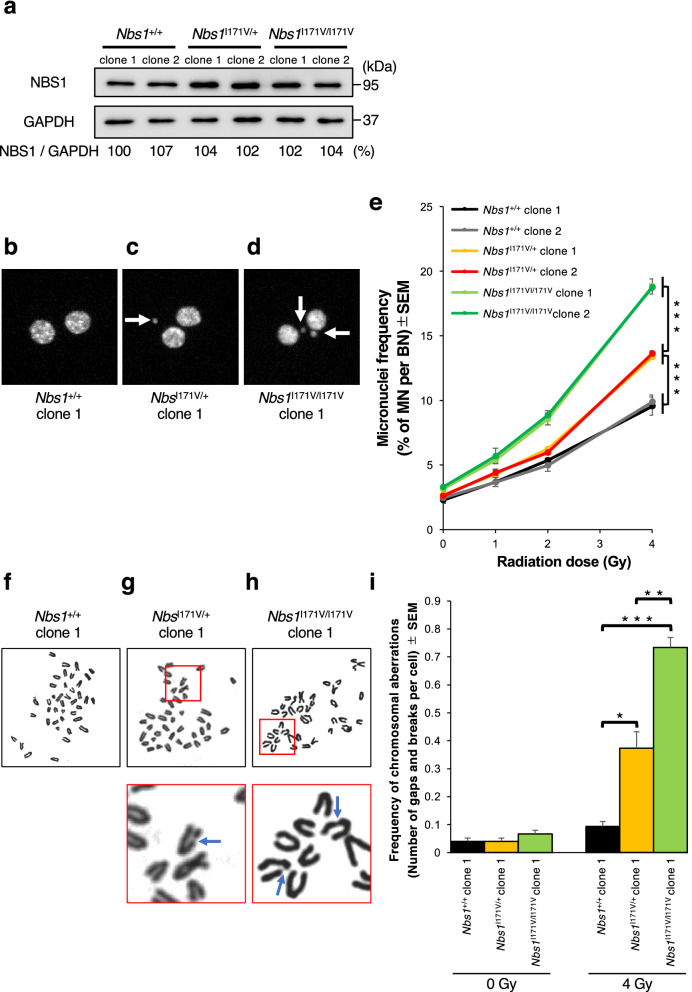
Radiation-induced chromosomal instability of *Nbs1* I171V edited MEF clones. (**a**) Western blotting analysis data showing the expression levels of Nbs1 protein in *Nbs1* I171V edited MEF clones. Full-length gel is presented in Supplementary Fig. 5. The GAPDH antibody was used as a loading control. The intensity of *Nbs1* bands was normalized to that of GAPDH and shown as a percentage, regarding the score of *Nbs1*^+/+^ clone 1 as 100%. (**b**–**d**) Metafer MN Search images showing the cytokinesis-blocked *Nbs1* I171V edited MEFs stained with DAPI. Arrowheads indicate MN. *Nbs1*^+/+^-BN cell without MN (**b**), *Nbs1*^I171V/+^-BN cell with one MN (**c**), *Nbs1*^I171V/I171V^-BN cell with three MN (**d**). (**e**) Percentage of IR-induced MN formation in *Nbs1* I171V edited MEF clones (mean ± SEM; *t*-test; n = 3; > 1000 BN cells). (**f, g**) Representative metaphase of *Nbs1* I171V-edited MEFs after 4 Gy irradiation. Remarkable aberrations are enlarged. Arrows indicate chromosomal breakages. (**i**) Frequency of IR-induced chromosomal aberrations in *Nbs1* I171V edited MEF clones (mean ± SEM; *t*-test; n = 3; > 50 cells).

To compare chromosomal radiosensitivity among the *Nbs1*^+/+^, *Nbs1*^I171V/+^, and *Nbs1*^I171V/I171V^ mice, we intercrossed the *Nbs1*^I171V/+^ mice to generate MEFs with each *Nbs1* genotype (Fig S1b–d). Western blotting analysis revealed that both *Nbs1*^I171V/+^ MEFs and *Nbs1*^I171V/I171V^ MEFs showed almost the same amount of Nbs1 protein as the *Nbs1*^+/+^ MEFs (Fig. 3a). Next, to quantify the effect of the copy number of the *Nbs1* I171V allele on chromosomal radiosensitivity, we measured the MN frequencies post-IR in these MEF lines using the semiautomatic CBMN assay (Fig. 3b–d). The *Nbs1*^I171V/I171V^ MEFs showed the highest rate of IR-induced micronucleus formation, and the *Nbs1*^I171V/+^ MEFs demonstrated a moderately radiosensitive phenotype in comparison with the *Nbs1*^+/+^ clones (Fig. 3e, S4a-c, Table S4). We also used Cabas software to evaluate the dose–response curves of the *Nbs1* mutant MEFs (Table 4). The mean data scores (D = 1 Gy) suggested that one copy of the *Nbs1* I171V allele contributed to an approximately 1.1-fold increase and two copies an approximately 1.7-fold increase of radiosensitivity compared with that of the biallelic wild-type *Nbs1*. Cytogenetic analysis also demonstrated that IR-induced chromosomal aberrations increased in a manner dependent on the copy number of the *Nbs1* I171V allele (Fig. 3f–i, S4d–f, Table S5). Taken together, these findings suggest that the *NBS1* c.511A>G, p.Ile171Val variant might be a genetic factor underlying individual differences in chromosomal radiosensitivity within human populations.

**Table 4:** Radiosensitivity coefficients (α, β, c, and α + β) in *Nbs1* I171V variant knock-in MEFs.

Cell line ID/genotype	β ± SE × 10^–3^	α ± SE × 10^–3^	c ± SE × 10^–3^	Radiosensitivity score [α + β]
	(Gy^-2^)	(Gy^-1^)		
*Nbs1*^+/+^ clone 1	0.997 ± 0.77	14.48 ± 1.92	22.56 ± 2.61	15.48 ± 1.17
*Nbs1*^+/+^ clone 2	2.576 ± 0.70	8.023 ± 2.86	24.83 ± 0.94	10.60 ± 2.25
*Nbs1*^I171V/+^ clone 1	3.960 ± 0.58	10.46 ± 1.70	27.34 ± 1.72	14.42 ± 1.30
*Nbs1*^I171V/+^ clone 2	4.261 ± 0.47	9.662 ± 2.44	26.94 ± 2.46	13.92 ± 1.97
*Nbs1*^I171V/I171V^ clone 1	5.840 ± 1.18	15.71 ± 4.20	31.75 ± 1.70	21.55 ± 3.09
*Nbs1*^I171V/I171V^ clone 2	5.148 ± 1.63	17.97 ± 6.28	33.09 ± 1.00	23.12 ± 4.65

α, β, and c coefficients were extracted from dose–response calibration curves in CBMN assay using Cabas software. Relative capacity to repair DNA after acute γ-irradiation was assessed at a dose of 1 Gy.

## Discussion

*NBS1* encodes the NBS1 protein, which consists of 754 amino acids and plays a central role in DNA DSB repair as a member of the MRN protein complex, which recognizes DNA DSB sites and orchestrates the DNA damage response^26^. NBS1 protein interacts with multiple phosphorylated DNA repair proteins through the FHA/BRCT domains (a forkhead‐associated domain and two BRCA1 C‐terminal domains) and is recruited to DNA DSB sites. The *NBS1* I171V variant, which was identified in this study, is located in the BRCT1 domain (Fig. 1a)^17^. It was reported that the overexpression of the NBS1 I171V protein in HeLa cells interfered with phosphorylated MDC1 and inhibited IR-induced formation of foci of NBS1^27^. Although overexpression analyses of the mutant protein are quick and informative, it is more precise to evaluate its effect on chromosomal radiosensitivity under the endogenous and physiological expression level. Consistent with previous overexpression studies, the endogenous promoter driven NBS1 I171V protein in HCT116 cells and MEFs also interfered chromosomal stability and cellular survival post IR irradiation. These results suggested that the *NBS1* I171V variant might be involved in individual radiosensitivity within human populations. However, the epidemiological cancer risk of this variant remains controversial. Therefore, we attempted to evaluate the precise effect of this variant on chromosomal radiosensitivity in a uniform genetic background.

In the presence of conflicting forward genetics data, it is difficult to evaluate whether the variants of interest are indeed involved in chromosomal radiosensitivity. The number of similar situations is increasing with the recent clinical sequencing using deep-sequencers for genetic diagnosis^28,29^. There are several criteria for evaluating the pathological significance of variants. We propose that a reverse genetics approach could be useful because this directly investigates the causality. In this study, we used the CRISPR/Cas9 system to introduce the *NBS1* I171V variant into human cultured HCT116 cells and mouse fertilized eggs. The biallelic *NBS1* I171V variant in human and mouse genomes significantly enhanced IR-induced micronucleus formation and chromosomal aberrations, clarifying that this variant underlies chromosomal instability after IR irradiation.

In general, simple knock-out using the CRISPR/Cas9 system can be applied in any proliferative human cultured cell lines. Previously, we generated *ATM* heterozygous knock-out hTERT-RPE1 cells derived from normal retinal tissues to demonstrate that the *ATM* heterozygous mutations affect cellular radiosensitivity^8^. The activity of homologous directed recombination (HDR) repair in human cultured cells from normal tissues is generally insufficient for ssODN-mediated knock-in. Therefore, here we used HCT116 cells with sufficient HDR activity and a diploid karyotype from colon cancer tissue. As expected, we developed two independent HCT116 cell clones with the biallelic *NBS1* I171V variant. However, *NBS1*^I171V/+^ cells were not obtained because the ssODN-non-targeted allele contained indel mutations in the CRISPR/Cas9 system-recognition site; however, mosaic colonies of *NBS1*^+/+^ cells and *NBS1*^I171V/ I171V^ cells were formed. To control the copy number of the *NBS1* I171V variant in the genome and to confirm that the biological effects of the variant on radiosensitivity are conserved among species, we generated mice heterozygous for the *Nbs1* I171V variant. Intercrossing of their heterozygous mice enabled MEFs with three copy numbers of the variant to be obtained. Notably, IR-induced chromosomal instabilities in the MEFs increased in a manner dependent on the copy number of the *Nbs1* I171V variant. Unlike a human *NBS1* I171V homozygote, the homozygous mice did not show aplastic anemia. Although heterozygotes of *NBS1* I171V are estimated to exist at a rate of approximately 0.15% in human populations (Table 2), a second homozygote has not been reported, implying that the existence of other gene mutations underlying aplastic anemia in the reported homozygote cannot be completely ruled out. Importantly, *NBS1* 171V did not increase the spontaneous frequencies of MN in HCT116 cells and MEFs in the spite of *NBS1* I171V carriers have significantly increased risk for multiple cancers even they are not radiated. Since NBS1 participates is not only DNA DSB repairs but also other DNA damage responses including Rad18/Polη-dependent translation DNA synthesis^30^ and RNF20-meditaed chromatin remodeling^31^, *NBS1* I171V might influence the genomic integrity against a variety of genotoxins to enhance the spontaneous carcinogenesis risk the *NBS1* I171V carriers. Further investigations are needed to confirm whether the heterozygous and homozygous *Nbs1* I171V exhibit a predisposition for cancer with or without IR irradiation, and to elucidate how *NBS1* I171V protein interferes the DNA damage response after IR irradiations. Taking the above findings together, it is safe to conclude that the *NBS1* I171V variant is a genetic factor underlying individual differences of radiation-induced chromosomal instability at the cellular level.

A series of radiation biological and epidemiological studies has suggested that many variants in the DNA repair genes might contribute to the existence of a portion of radiosensitive populations^32^. Besides the *NBS1* I171V variant, here we also extracted several suspicious variants in the *ATM* and *TP53* genes from Japanese ovarian cancer patients. Whether they are merely polymorphisms or indeed determinants of IR-induced chromosomal instability and carcinogenesis is a key question for precision medicine in the fields of radiology and oncology. Indeed, in breast cancer patients with variants such as in *BRCA1*, *BRCA2*, and *ATM*, it is recommended that the contralateral breast dose in mammography and radiotherapy be minimized in order to prevent secondary carcinogenesis^33^. Since chromothripsis, which is massive and clustered genomic rearrangements, occurs in micronuclei to drive cancer evolution^34,35^, the frequent radiation-induced micronucleus formation in the carriers with *NBS1* I171V and other DNA repair gene variants might enhance the risk of secondary carcinogenesis. Thus, knowledge of the genetic basis underlying individual differences in chromosomal radiosensitivity might allow us to provide safer approaches in the context of CT imaging and radiotherapy.

Although the reverse genetics approach used in this study is useful for precision medicine, the throughput should be improved. Recent advances of CRISPR/Cas systems provide the base-editor technology based on several cytidine deaminases and their chemical evolved products fused to catalytically dead Cas9 (dCas9), thereby converting the base pair at the specific site directly^36^. Prime editing technology based on dCas9 fused with an engineered reverse transcriptase is another potential approach of base pair conversion^37^. If the base-editor and prime editing technologies are applied to the human haploid cell line HAP1 derived from chronic myeloid leukemia^38^, the higher-throughput validation of single-nucleotide variants by reverse genetics might provide convincing genetic markers for personal radiological protection and accurate genetic diagnosis.

## Materials and methods

### Whole-exome analysis

This study was reviewed and approved by the Ethics Committee of Hiroshima University Hospital, and conducted in accordance with the Declaration of Helsinki. Written informed consent was obtained from the participants. Genomic DNA was extracted from the peripheral lymphocytes of ovarian cancer patients and exonic DNA was captured using Agilent SureSelect Human All Exon V5 kit. Sequencing was performed with 150-bp paired-end reads on a HiSeq2500 (Illumina). We used BWA (http://bio-bwa.sourceforge.net/) for alignment and mapping, Samtools (http://samtools.sourceforge.net/) and Picard (http://broadinstitute.github.io/picard/) for SAM/BAM handling, GATK (http://www.broadinstitute.org/gatk/) and Samtools for variant calls, and Annovar (http://annovar.openbioinformatics.org/) for annotation, as described previously. Functional predictions associated with amino acid changes were performed using PolyPhen-2 (http://genetics.bwh.harvard.edu/pph2) and MutationTaster (http://www.mutationtaster.org/index.html). All reported genomic coordinates were in the Genome Reference Consortium Human build 37 (GRCh37/hg19) assembly. PCR amplification followed by Sanger sequencing with an Applied Biosystems 3130 sequencer (ThermoFisher) was used to validate mutations identified by whole-exome sequencing.

### Cell cultures

HCT116 cells were cultured in Dulbecco’s modified Eagle’s medium (DMEM) supplemented with 10% fetal bovine serum (FBS) and gentamycin at 37 °C in humidified air with 5% CO_2_. Primary MEFs were cultured in DMEM supplemented with 20% FBS and penicillin/streptomycin at 37 °C in humidified air with 5% CO_2_.

### Animals and generation of MEFs

This study was reviewed and approved by the Ethics Committee for Experimental Animals of Hiroshima University, and all animals were treated in accordance with the guidelines of the Institutional Animal Care and Use Committee. The E13.5 embryos were thoroughly minced with razor or scalpel blades. The cells spun down at 500×*g* for 5 min were incubated in DMEM supplemented with 20% FBS and penicillin/streptomycin at 37 °C in humidified air with 5% CO_2_. After 24 h, a resuspension of the 2.5% trypsin/EDTA (Gibco)-treated cells was filtrated through a 40 µm cell strainer and subsequently centrifuged at 500×*g* for 5 min. The cellular pellet was resuspended in DMEM supplemented with 20% FBS and penicillin/streptomycin and cultured in 60-mm culture dishes.

### Generation of *NBS1* I171V HCT116 knock-in genome-edited cells

A total of 200 pmol 100-mer ssODNs carrying the *NBS1* c.511 A>G, p.I171V variant and a silent ScaI site (5′-CCTTTCAATTTGTGGAGGCTGCTTCTTGGACTCAACTGCTTTCAGGAATTCAGTAAAgTAcTCcGGCTTcACAACTGGACGTCCACAAATGAGTGCACATATTGTCTACAATGAAGAAAACATGTGAATATATATATTCACATGCTAGCA-3′) or the silent mutations (5′- CCTTTCAATTTGTGGAGGCTGCTTCTTGGACTCAACTGCTTTCAGGAATTCAGTAAAgTAcTCcGGCTTcACAATTGGACGTCCACAAATGAGTGCACATATTGTCTACAATGAAGAAAACATGTGAATATATATATTCACATGCTAGCA-3′) and 1 μg of the pX459 plasmid vector [pSpCas9(BB)-2A-Puro; Addgene plasmid #62988] for editing of exon 5 of the *NBS1* gene were cotransfected into 1 × 10^6^ HCT116 cells with Kit V (Lonza) and program D-032 (HCT116) (Nucleofector™ 2b device; Lonza), in accordance with the manufacturer’s protocol. Twenty-four hours after transfection, cells were treated with 1 μg/ml puromycin (Nacalai Tesque) for 48 h. After 2 weeks, the drug-resistant colonies were isolated and divided into two aliquots: one was transferred into a well of a 96-well plate for clonal expansion, while the other was lysed and used for PCR with a pair of primers for the amplification of *NBS1* exon 5 (forward primer 5′-ATCGGTGACTTCTTATAATTGAGTG-3′-, and reverse primer 5′--GCGTGATGCCTGGAACTGACATATG-3′-). For the screening of *NBS1*^I171V/I171V^ cells and *NBS1*^+/+^ cells with the silent mutations, PCR fragments were digested with *Sca*I and then run on 2.0% agarose gel. The *Sca*I-sensitive products were analyzed by direct-sequence genotyping (ABI 3130 sequencer).

### Generation of *Nbs1* I171V knock-in genome-edited mice

C57BL6 mouse fertilized eggs were electroporated with recombinant Cas9 protein (GeneArt Platinum Cas9 Nuclease, ThermoFisher), chemically synthesized *NBS1-*crRNA (5′-cauuaugaguccuuaagugaGAATACTTTTCTGAATTTCT-3′, Fasmac), chemically synthesized tracrRNA (5′-AAACAGCAUAGCAAGUUAAAAUAAGGCUAGUCCGUUAUCAACUUGAAAAAGUGGCACCGAGUCGGUGCU-3′, Fasmac), and ssODN carrying *Nbs1* I171V and *Eco*RI enzyme digestion sequence (silent mutations) using NEPA21 (NEPAGENE). These eggs were transferred into pseudo-pregnant female mice. Genomic DNA from the tail of F_0_ generation mice was analyzed by PCR with a pair of forward/reverse primers for the amplification of *Nbs1* exon 5 (forward primer 5′-TGGACTAGGGTCTCTGTCGCTCTTGAATAAGCTGTTCTACAGAT-3′, and reverse primer 5′-CGCTCCTAGGTCTCACGGTAACCAACACCTACTCATTTCTATGC-3′). PCR products were checked by *Eco*RI enzyme digestion (New England Biolabs) and DNA direct sequencing (ABI 3130 sequencer). The ssODN knock-in male mice were mated with C57BL6 females and the offspring were genotyped to confirm the germline transmission of the ssODN knock-in allele. Heterozygous knock-in mice were then intercrossed to produce homozygous mice. All animal methods were performed in accordance with the ARRIVE guidelines.

### Western blotting analysis

Cells were resuspended in lysis buffer (20 mM Tris–HCl pH 8.0, 150 mM NaCl, 2 mM EDTA, 0.1% SDS, 1% NP-40, 0.1% sodium deoxycholate, protease inhibitors, and 10 mM NaF) and incubated on ice for 20 min. Samples were centrifuged at 8000×*g* for 20 min and the supernatants were collected. Protein concentration in the supernatant was determined by Bradford protein assay. Cell lysates were then boiled with sample buffer (60 mM Tris–HCl pH 6.8, 1% SDS, 5% β-mercaptoethanol) for 5 min. Proteins were separated via SDS-PAGE and transferred to a PVDF membrane. The membrane was blocked with 5% skim milk in TBS-T (0.05% Tween-20) for 30 min. Primary antibodies were diluted in blocking buffer and the membrane was incubated with diluted antibodies overnight at 4 °C. HPR-conjugated anti-rabbit and anti-mouse IgG (#NA934V and #NA931V, respectively; GE Healthcare) were used as secondary antibodies. Images were acquired using ImageQuant LAS4000mini system (GE Healthcare). Quantitation of the levels of NBS1, MRE11 and GAPDH was performed by ImageQuant TL 7.0 software version 8.1 (GE Healthcare). The primary antibodies used were anti-NBS1 mouse monoclonal antibody (GTX70222, GeneTex and MAB15731, R&D), anti-MRE11 rabbit polyclonal antibody (ab33125, Abcam) and anti-GAPDH mouse monoclonal antibody (sc-32233, Santa Cruz Biotechnology).

### RT-PCR

RT-PCR analysis was carried out as described previously^39^. Briefly, HCT116 genome editing cells in 96-well plates were cultured in DMEM supplemented with 10% FBS for 24 h. The cells were washed with ice-cold PBS, lysed, and then, cDNAs were synthesized with reverse transcriptase from their extracted total RNA using Cells to CT Kit (ThermoFisher), in accordance with the manufacturer's protocol. Expression was measured by TaqMan PCR analysis using *MRE11* and *GAPDH* TaqMan probes (ThermoFisher) on a CFX Connect™ RealTime PCR Detection System (BioRad); the levels of *MRE11* expression were normalized to those of *GAPDH*. The primers and TaqMan probes were *MRE11* (Hs00967437_m1) and *GAPDH* (Hs99999905_g1).

### Gamma irradiation

Cells were irradiated from 1 to 4 Gy (^137^Cs gamma ray source, 148 TBq, Gammacell 40 Exactor; Best Theratronics). The dose rate used was around 1 Gy/min.

### Semiautomatic cytochalasin-block micronucleus (CBMN) assay

Cells were seeded in six-well plates, incubated for 4 h, and then irradiated. After 44 h of incubation, 3 μg/ml cytochalasin B (WAKO) was added to block cytokinesis for 24 h. Cells were harvested and centrifuged. The pellet was treated with hypotonic solution (75 mM KCl) for 15 min at 37 °C and subsequently rinsed in Carnoy solution (methanol: acetic acid, 3:1) three times for fixation. The slides were prepared using HANABI Metaphase Spreader (ADSTEC, Japan) and stained with 1 μg/ml 4′,6-diamidino-2-phenylindole (DAPI). These cells were scanned at 10 × magnification with a Metafer 4 Scanning System comprising a Carl Zeiss Axioplan Imager Z1 connected to Metafer 4 software version 3.11.4 (MetaSystems GmbH, Altlussheim Germany). Captured images were analyzed with the Metafer 4_MN program (MetaSystems). Criteria for selecting BN cells and MN were as previously described^8^. All scanned images of BN cells and MN were re-evaluated visually to exclude false-positive and -negative images of BN cells and for MN to be scored in at least 1000 BN cells from each condition.

### Survival assay

An appropriate number of cells were irradiated and then seeded in three 100-mm dishes per condition. After 11 days of incubation, cell colonies were fixed with 100% methanol at room temperature for 2 min and stained with 10% Giemsa (Wako) for 2 min. Survival curves were obtained by counting the number of cell colonies.

### Chromosomal aberration analysis

Cells were seeded in six-well plates, incubated for 4 h (HCT116 cells) or 6 h (MEFs), and then irradiated. After 24 h of incubation, colcemid (0.1 mg/ml, Gibco) was added to induce arrest in metaphase for 1 h (HCT116 cells) or 6 h (MEFs). The cells were then treated with a hypotonic solution (0.075 M KCl) for 15 min at 37 °C and subsequently fixed with Carnoy solution. Slides were prepared using HANABI Metaphase Spreader (ADSTEC, Japan) and then stained with 10% Giemsa solution (Wako) for 5 min. Chromosome images were scanned at 10 × and 63 × magnifications with Metafer 4 software (MetaSystems, Germany) connected to a motorized ZEISS AxioImager M1 (Zeiss, Germany). Chromosome gaps and breakages were scored visually by fewer than 25 metaphases per slide of well-spread metaphase chromosomes. Fifty metaphase chromosomes from each condition were examined for spontaneous chromosomal aberrations.

### Statistical analysis

The experiments were performed independently three times, and the data are shown as mean ± s.e. Differences between groups were evaluated for statistical significance using Student’s *t*-test. Values of p < 0.05 were considered to be statistically significant.

## Supplementary Information

Supplementary Information.
