# Supplementary material for: NBS1 I171V variant underlies individual differences in chromosomal radiosensitivity within human populations
Source: Sci Rep. 2021 Oct 4;11:19661. doi: 10.1038/s41598-021-98673-7 (PMC8490386; doi:10.1038/s41598-021-98673-7)
Supplement: Supplementary file 1 — Supplementary Information. [file 41598_2021_98673_MOESM1_ESM.pdf]

Supplementary Information

Figures S1 to S5 and Tables S1 to S5

NBS1 I171V variant underlies individual differences in chromosomal radiosensitivity within human populations

Keita Tomioka[1,2,#], Tatsuo Miyamoto[1,#], Silvia Natsuko Akutsu[1], Hiromi Yanagihara[1], Kazumasa Fujita[1], Ekaterina Royba[1,3], Hiroshi Tauchi[4], Takashi Yamamoto[5], Iemasa Koh[6], Eiji Hirata[6], Yoshiki Kudo[6], Masao Kobayashi[2], Satoshi Okada[2], and Shinya Matsuura[1,*]

[1]*Department of Genetics and Cell Biology, Research Institute for Radiation Biology and Medicine, Hiroshima University, Hiroshima 734-8553, Japan*
[2]*Department of Pediatrics, Graduate School of Biomedical and Health Sciences, Hiroshima University, Hiroshima 734-8551, Japan*
[3]*Center for Radiological Research, Columbia University Irving Medical Center, New York, 10032, USA*
[4]*Department of Biological Sciences, Faculty of Science, Ibaraki University, Mito 310-8512, Japan*
[5]*Program of Mathematical and Life Sciences, Graduate School of Integrated Sciences for Life, Hiroshima University, Higashi-Hiroshima 739-8526, Japan*
[6]*Department of Obstetrics and Gynecology, Graduate School of Biomedical and Health Sciences, Hiroshima University, Hiroshima 734-8551, Japan*

[#]These authors contributed equally to this work.
Correspondence: e-mail: shinya@hiroshima-u.ac.jp

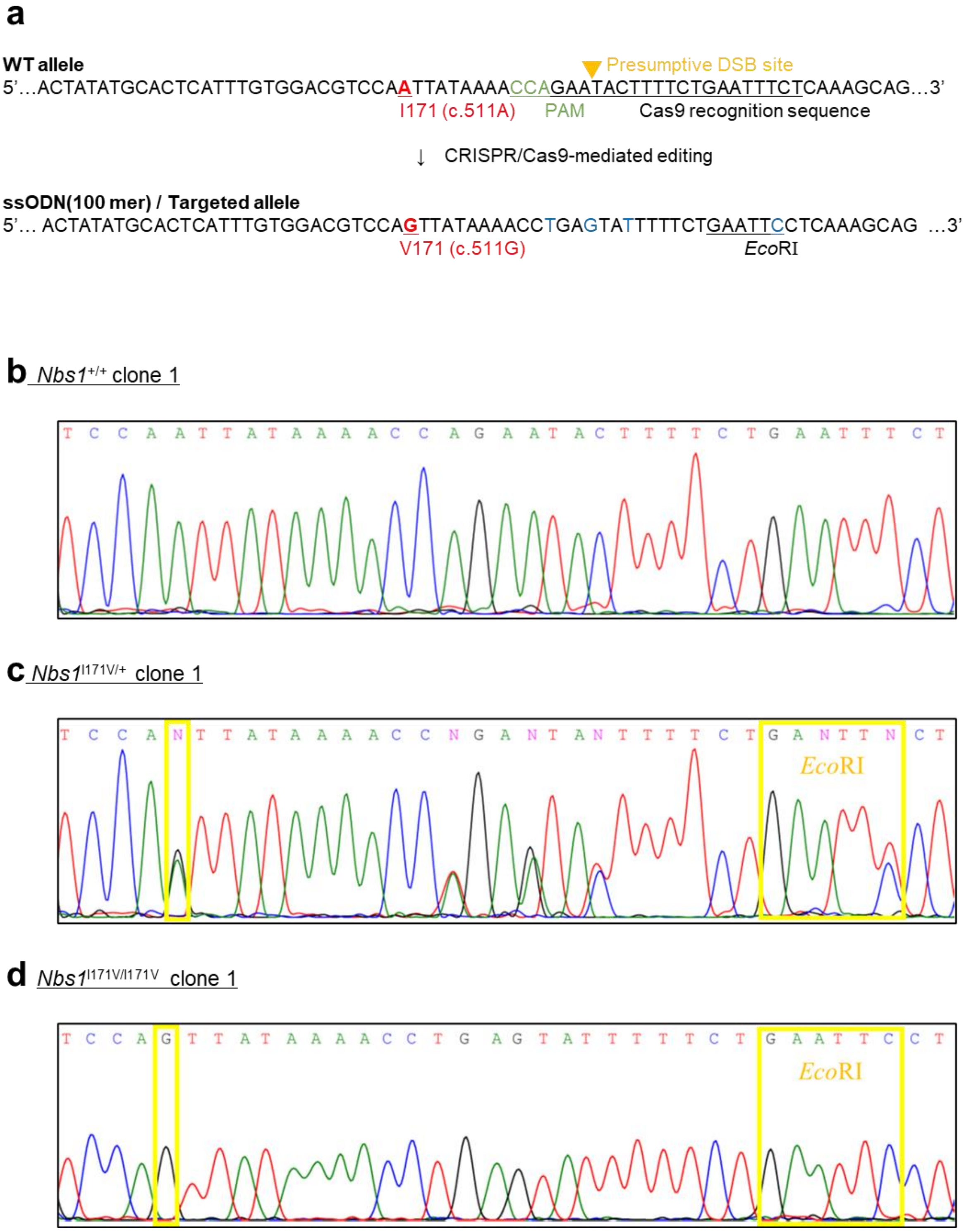

Figure S1. Generation of *Nbs1* I171V knock-in mice.

(a) Targeting strategy for *Nbs1* I171V knock-in using the CRISPR/Cas9 system. Blue bases indicate silent mutations.

(b–d) Sanger sequencing of *Nbs1*$^{+/+}$-MEF clone 1 (b), *Nbs1*$^{I171V/+}$-MEF clone 1 (c), and *Nbs1*$^{I171V/I171V}$-MEF clone 1 (d). A single base substitution of the *Nbs1* I171V variant and a silent *Eco*RI site are indicated by yellow boxes.

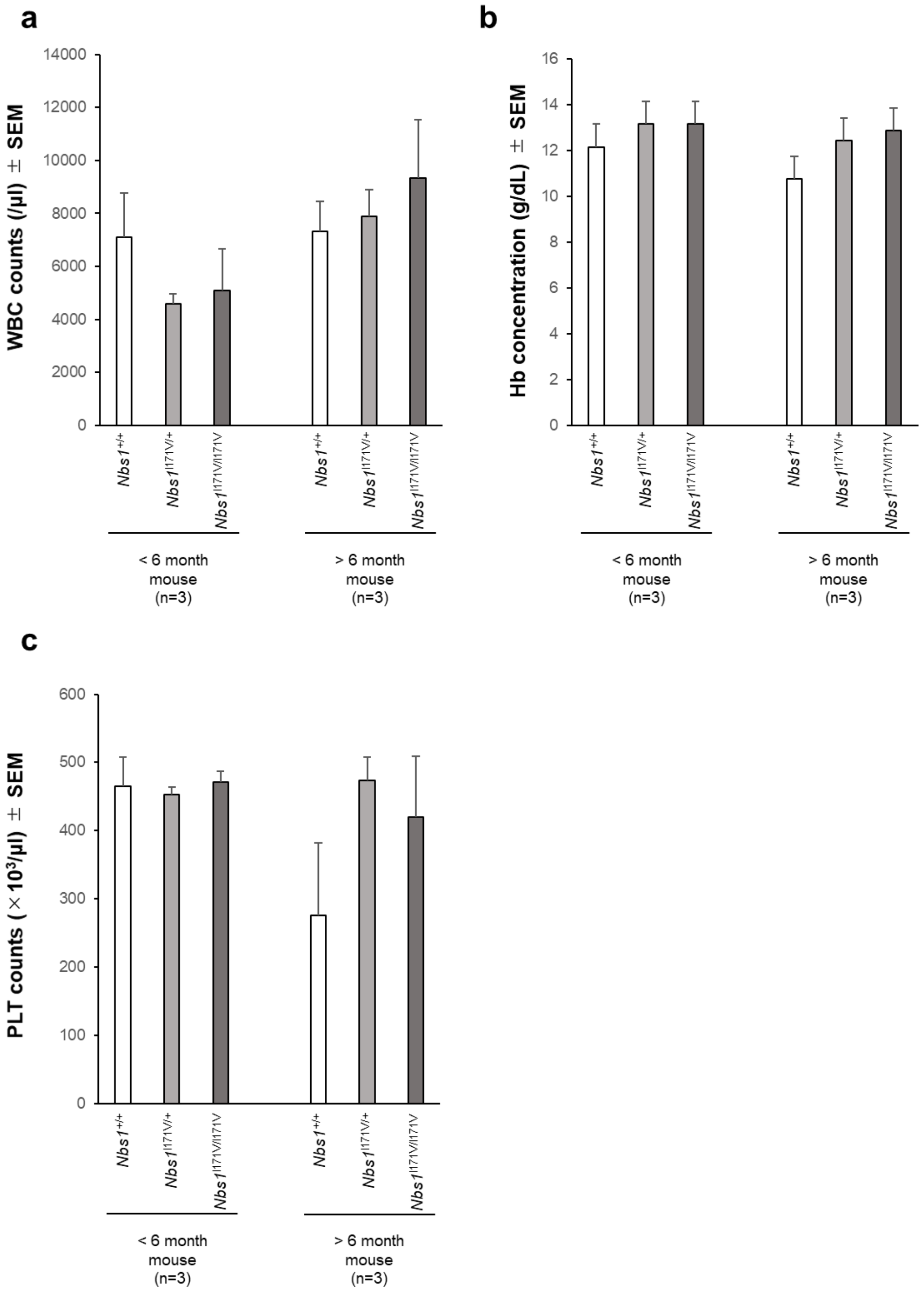

Figure S2. Complete blood counts in the *Nbs1* I171V knock-in mice.

(a) White blood cell (WBC) counts, (b) hemoglobin (Hb) concentrations, and (c) platelet (PLT) counts of $Nbs1^{+/+}$, $Nbs1^{I171V/+}$, and $Nbs1^{I171V/I171V}$ mice (mean±SEM; no significant change in each t-test parameter; n=3).

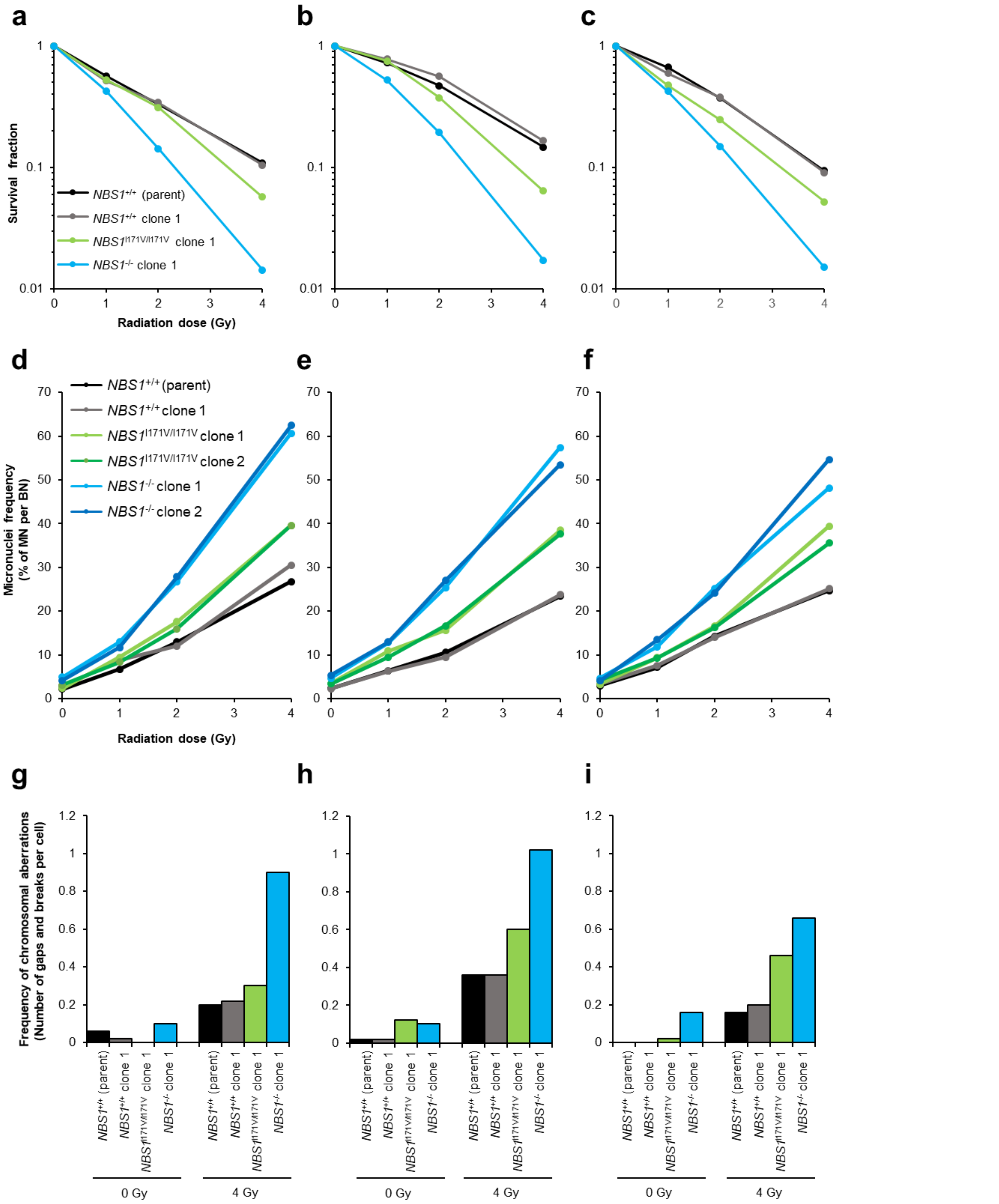

Figure S3. Three independent colony survival, CBMN, and chromosomal aberration assays in *NBS1* I171V knock-in HCT116 cell clones.

Three graphs showing the results of three independent experiments: survival fractions (a–c), IR-induced MN formation (d–f), and IR-induced chromosomal aberration (g–i).

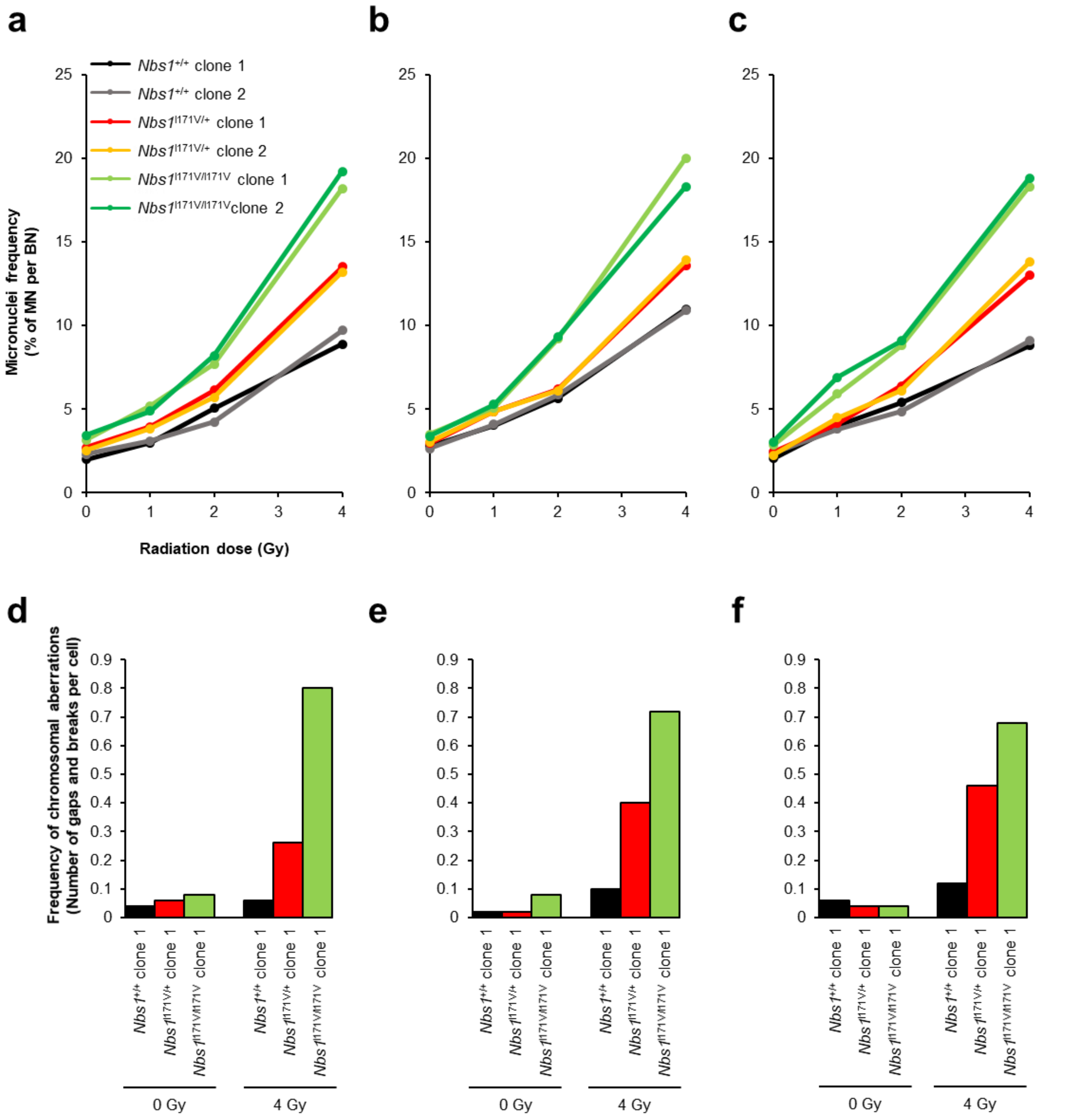

Figure S4. Three independent CBMN and chromosomal aberration assays in the *Nbs1* I171V knock-in MEF clones.

Three graphs showing the results of three independent experiments: percentage of IR-induced MN formation (a–c) and IR-induced chromosomal aberration (d–f).

NBS1 knock-in HCT116 cells

NBS1 95 kDa

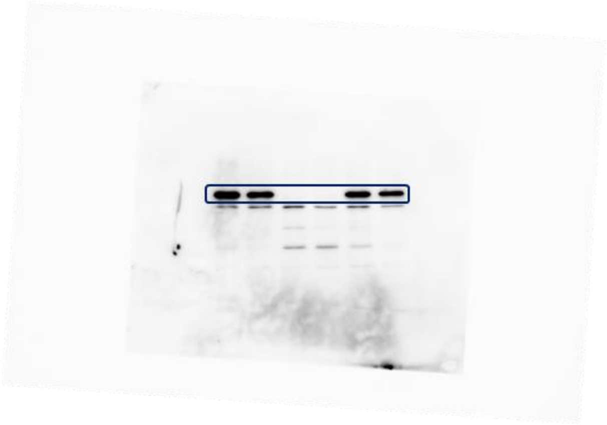

GAPDH 37 kDa

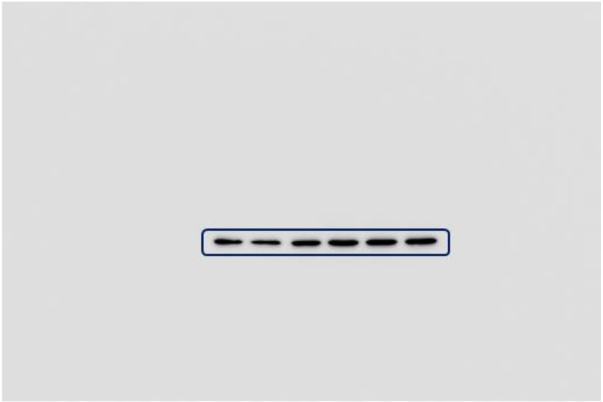

MRE11 81 kDa

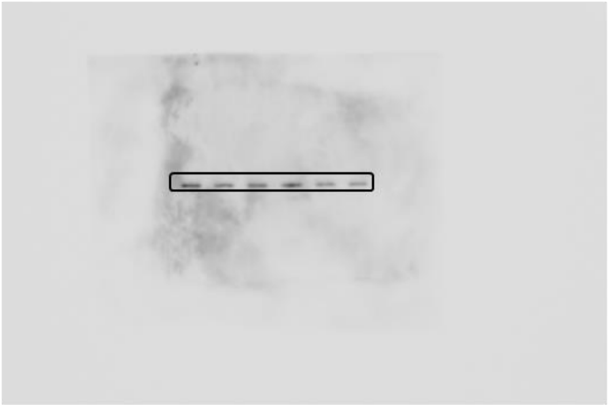

Nbs1 knock-in MEFs

NBS1 95 kDa

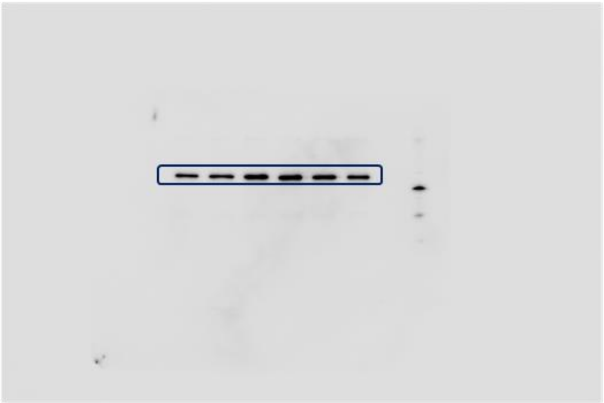

GAPDH 37 kDa

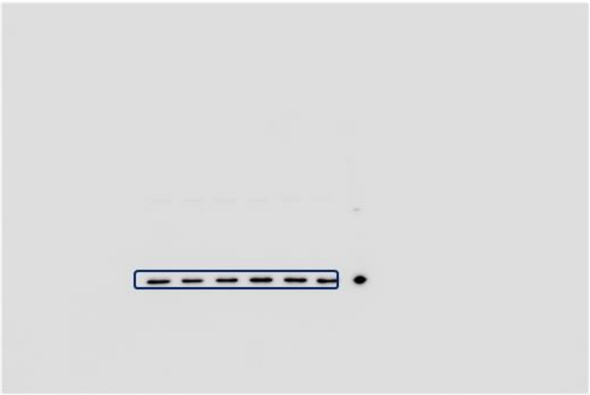

Figure S5. Uncropped images of western blotting data shown in the main text.

		1Gy	2Gy	4Gy
NBS1$^{+/+}$	*NBS1*$^{I171V/I171V}$	0.51	0.14	0.013
NBS1$^{+/+}$	*NBS1*$^{-/-}$	0.023	0.0027	0.00081
NBS1$^{I171V/I171V}$	*NBS1*$^{-/-}$	0.24	0.021	0.00029

Table S1. T-test of survival fraction in *NBS1* I171V knock-in HCT116 cell clones.

		0Gy	1Gy	2Gy	4Gy
NBS1$^{+/+}$	*NBS1*$^{I171V/I171V}$	0.061	0.00094	0.00047	0.0000014
NBS1$^{+/+}$	*NBS1*$^{-/-}$	0.000022	0.00000045	0.000000055	0.00000016
NBS1$^{I171V/I171V}$	*NBS1*$^{-/-}$	0.0014	0.000028	0.000000034	0.000011

Table S2. T-test of CBMN assay in *NBS1* I171V knock-in HCT116 cell clones.

		0Gy	4Gy
NBS1$^{+/+}$	*NBS1*$^{I171V/I171V}$	0.37	0.034
NBS1$^{+/+}$	*NBS1*$^{-/-}$	0.0010	0.00021
NBS1$^{I171V/I171V}$	*NBS1*$^{-/-}$	0.16	0.041

Table S3. T-test of Chromosomal aberration in *NBS1* I171V knock-in HCT116 cell clones.

		0Gy	1Gy	2Gy	4Gy
Nbs1$^{+/+}$	*Nbs1*$^{I171V/+}$	0.14	0.030	0.0045	0.0000052
Nbs1$^{+/+}$	*Nbs1*$^{I171V/I171V}$	0.00042	0.00043	0.0000015	0.000000052
Nbs1$^{I171V/+}$	*Nbs1*$^{I171V/I171V}$	0.0054	0.0070	0.0000027	0.000000013

Table S4. T-test of CBMN assay in *NBS1* I171V knock-in MEF clones.

		0Gy	4Gy
Nbs1$^{+/+}$	*Nbs1*$^{I171V/+}$	1	0.011
Nbs1$^{+/+}$	*Nbs1*$^{I171V/I171V}$	0.205106455	0.000084
Nbs1$^{I171V/+}$	*Nbs1*$^{I171V/I171V}$	0.205106455	0.0064

Table S5. T-test of Chromosomal aberration in *NBS1* I171V knock-in MEF clones.
